# Effect of spermidine intake on overload‐induced skeletal muscle hypertrophy in male mice

**DOI:** 10.14814/phy2.70209

**Published:** 2025-02-05

**Authors:** Tomohiro Iwata, Takanaga Shirai, Riku Tanimura, Ryoto Iwai, Tohru Takemasa

**Affiliations:** ^1^ Graduate School of Comprehensive Human Sciences University of Tsukuba Ibaraki Japan; ^2^ Research Fellow of Japan Society for Promotion Science Tokyo Japan; ^3^ Department of Human Sciences Kanagawa University Kanagawa Japan; ^4^ Institute of Health and Sport Sciences University of Tsukuba Ibaraki Japan

**Keywords:** autophagy, mitochondria, mTOR signaling, skeletal muscle hypertrophy, spermidine

## Abstract

Skeletal muscles exhibit high plasticity, such as overload‐induced hypertrophy or immobilization‐induced atrophy. During sports, skeletal muscle hypertrophy is induced by training to improve performance. Spermidine is a type of polyamine and oral intake of spermidine exerts many beneficial effects on health through various mechanisms, such as promoting autophagy and improving mitochondrial function. In a recent study, we showed that spermidine intake activates mTOR signaling and significantly increases the mean fiber cross‐sectional area (CSA) 14 days after injury. This suggests that spermidine promotes the anabolic growth of differentiated muscle (i.e., muscle hypertrophy); however, calorie restriction, which has been reported to have effects on the same molecular mechanisms as spermidine (promoting autophagy and improving mitochondrial function), promotes skeletal muscle regeneration, while inhibiting skeletal muscle hypertrophy. Therefore, we evaluated the effect of spermidine intake on skeletal muscle hypertrophy in mice using a synergistic ablation‐induced muscle hypertrophy model. Our results showed that spermidine intake significantly decreased mean myofiber of CSA, but this was not consistent with the change in skeletal muscle wet weight. We also analyzed autophagy, mTOR signaling, inflammation, and mitochondria, but no significant effects of spermidine intake were observed at most protein expression levels. Therefore, spermidine intake does not affect overload‐induced skeletal muscle hypertrophy, and even if it does, the effect is suppressive.

## 1 INTRODUCTION

Skeletal muscles are attached to bones via tendons and move joints by contracting, which enables physical movement. Skeletal muscle mass and cross‐sectional area (CSA) are determinants of the skeletal muscle contraction force and are important for athletic performance. Athletes constantly control their skeletal muscle mass by training and dietary management. Nutritional intake and mechanical stimulation by resistance training phosphorylate mTORC1 and its downstream target S6 (activating mTOR signaling). This increases protein synthesis in ribosomes and increases skeletal muscle mass. mTOR activates synthetic pathways while negatively regulating degradative pathways, such as autophagy (Atherton & Smith, 2012). Autophagy is an intracellular degradation mechanism that improves the intracellular environment and supplies energy by breaking down intracellular components and unneeded molecules (Galluzzi & Green, 2019). Under starvation conditions, such as calorie restriction, mTOR signaling is inactivated and autophagy is promoted, resulting in decreased skeletal muscle mass. (Nonaka et al., 2017).

Spermidine is a type of polyamine found in foods, such as soybeans or cheese, and has a wide range of health benefits. Previous studies indicate that spermidine intake suppresses immobilization‐induced skeletal muscle atrophy (Segales et al., 2020). Spermidine intake also improves skeletal muscle pathology caused by collagen VI deficiency and β‐galactose administration (Chrisam et al., 2015; Fan et al., 2017). We recently demonstrated that spermidine intake activates mTOR signaling and significantly increases the mean fiber CSA 14 days following injury. This suggests that spermidine may promote the anabolic growth of differentiated muscle (i.e., muscle hypertrophy) (Iwata et al., 2024).

Spermidine is also known as a calorie restriction mimetic because of its effects and mechanisms, such as promoting autophagy and improving mitochondrial function. Recent studies have reported that spermidine is essential for promoting autophagy and extending lifespan through calorie restriction (Ingram & Roth, 2015). Previous studies have reported that calorie restriction promotes skeletal muscle regeneration, similar to spermidine; however, calorie restriction inhibits skeletal muscle hypertrophy (Cerletti et al., 2012). However, previous research has shown that calorie restriction inhibits skeletal muscle hypertrophy (Shirai et al., 2022). However, no studies examine the effect of spermidine intake on skeletal muscle hypertrophy. Therefore, we evaluated the effect of spermidine intake on skeletal muscle hypertrophy using a synergist ablation‐induced muscle hypertrophy in mice.

## 2 MATERIALS AND METHODS

### 2.1 Animals

All experimental procedures performed in this study were reviewed and approved by the Institutional Animal Experiment Committee of the University of Tsukuba (animal ethical approval number 24–374) badsed on the guidelines of National Institutes of Health for Care and Use of Laboratry Animals (National Research Council Committee for the Update of the Guide for the Use of Laboratry 2011). Further, 7‐week‐old Male C57BL/6j mice (The Jackson Laboratory Japan, Inc., Kanagawa, Japan) were used in this study. The mice were maintained at a temperature of 22°C ± 2°C and a humidity of 55% ± 5% in controlled facilities under a 12/12 h light/dark cycle with ad libitum access to food (23.1% protein: 5.1% fat: 71.8% carbohydrate; Oriental, Japan) and distilled water. After 1 week of acclimatization, we performed synergist ablation surgeries under anesthesia with isoflurane (2.0%–3.0% isoflurane in air) inhalation as previously described (McCarthy et al., 2011) (Miyazaki et al., 2011). This in vivo model induces hypertrophy of the plantaris muscle through mechanical overload, resulting from the surgical removal of synergist muscles (gastrocnemius and soleus). This in vivo model induces hypertrophy of the plantaris muscle through mechanical overload, resulting from the surgical removal of the synergistic muscles (gastrocnemius and soleus). Grouping was performed immediately after synergist ablation surgeries. The mice were randomly assigned to groups. The DW group was provided access to distilled water ad libitum and the SP group was provided access to a 5 mM concentration of spermidine (14,918; CAY) mixed with distilled water ad libitum (Ito et al., 2021). As a normal control, the group had access to distilled water ad libitum without synergist ablation surgeries (*n* = 6 per group). After 4 and 14 days, the mice were euthanized by cervical dislocation and the plantaris muscle was excised, weighed, flash‐frozen in liquid nitrogen or liquid nitrogen‐cooled isopentane, and stored at −80°C.

### 2.2 Western blot analysis

Dissected plantaris muscles were immediately frozen in liquid nitrogen and total muscle protein was extracted by homogenizing in 50 mM HEPES (pH 7.6), 150 mM NaCl, 10 mM EDTA, 10 mM Na_4_P_2_O_7_, 10 mM NaF, 2 mM Na_3_VO_4_, 1% (v/v) NP‐40, 1% (v/v) Na‐deoxycholate, 0.2% (w/v) SDS, and 1% (v/v) complete protease inhibitor cocktail (169–26,063; Wako). The protein concentrations were measured using the Protein Assay Bicinchoninate Kit (297–73,101; Wako). Prior to SDS‐PAGE separation, an aliquot of the extracted protein solution was combined with an equal volume of sample loading buffer consisting of 1% (v/v) 2‐mercaptoethanol, 4% (w/v) SDS, 125 mM Tris–HCl (pH: 6.8), 10% (w/v) sucrose, and 0.01% (w/v) bromophenol blue and heated at 37°C for 1 h. The protein samples (10 μg) were separated by SDS‐PAGE and transferred to an ImmunoBlot PVDF membrane (#1620177; Bio‐Rad Laboratories). The blots were blocked for 1 h at room temperature in a blocking solution [5% skim milk (Yukijirushi), 5% Blocking One (03953–95; Nacalai Tesque, Inc.) containing 0.1% Tween‐20 in TBS] followed by primary antibodies in TBS containing 0.1% Tween‐20 and incubated overnight at 4°C. After incubation, the membranes were incubated with a horseradish peroxidase‐conjugated secondary antibody, anti‐rabbit IgG (7074P2; CST), anti‐mouse IgG (7076P2; CST), or anti‐goat IgG (HAF017; R&D Systems) for 60 min at room temperature. The signals were detected using Immunostar Zeta or LD (295–72,404/299–69,904; Wako Chemicals, Osaka, Japan), quantified with C‐Digit (LI‐COR Biosciences, Lincoln, NE, USA), and expressed in arbitrary units. Coomassie brilliant blue staining was used to confirm equivalent loading.

### 2.3 Primary antibodies for western blot analysis

The following primary antibodies were used for western blot analysis: anti‐MAP1LC3 Microtubule‐associated protein 1 light chain 3 (LC3) (4108; CST), anti‐p62 (SQSTM1) (PM045; MBL), anti‐ubiquitin (sc‐166,553; Santa Cruz), anti‐eIF4E (C46H6, #2067; CST), anti‐p‐eIF4E (S209, #9741; CST), anti‐rpS6 (#2217; CST), anti‐p‐rpS6 (Ser240/244, #5364P; CST), anti‐p‐rpS6 (Ser235/236; #4858S; CST), anti‐p‐P70S6 kinaseα (E‐8) (sc‐377,529; Santa Cruz), anti‐p70S6 Kinase (#9202; CST), anti‐oxidative phosphorylation (OXPHOS) (ab110413; abcam), anti‐cytochrome c (ab13575; abcam), anti‐PGC‐1α (AB3242; Millipore), anti‐TNF‐α (TN3‐19, 12) (sc‐12,744; Santa Cruz), and anti‐IL‐6 (AF406NA; R&D Systems).

### 2.4 Immunohistochemistry and CSA analysis

The plantaris muscles were covered with an optimal cutting temperature compound (4583; Sakura Finetek, Tokyo, Japan), frozen in liquid nitrogen‐cooled isopentane, and stored at −20°C until sectioning. The frozen plantaris muscles were sliced into 10‐μm sections using a cryostat (NX70; ThermoFisher Scientific K.K., Tokyo, Japan), fixed in 4% paraformaldehyde, permeabilized with 0.1% Triton X‐100, and blocked with M.O.M. blocking reagent (2 drops in 1.25 mL PBS) (MKB‐2213; Vector Laboratories, Newark, CA, USA). For muscle fiber type analysis, the fixation and permeabilization steps were not performed and the tissue was blocked with M.O.M. blocking reagent (1% in PBS) for 1 h. The sections were incubated with primary antibodies overnight at 4°C followed by secondary antibodies for 1 h at room temperature in the dark. For CSA analysis, the antibodies were diluted with 0.1% TritonX‐100, 1% horse serum (12,449; JRH biosciences) in PBS. The primary antibody was rat anti‐laminin α‐2 (4H8‐2; sc‐59,854; Santa Cruz) and the secondary antibody was goat anti‐rat IgG Alexa Fluor‐546 (A11035; Invitrogen). For muscle fiber type analysis, the antibodies were diluted1:30 with M.O.M. protein concentrate (BMK‐2202; Vector Laboratories) in PBS and rat anti‐laminin α‐2 (4H8‐2, sc‐59,854; Santa Cruz), Mouse IgG2b Myosin heavy chain Type I (BA‐D5; DSHB), Mouse IgG Myosin heavy chain Type II A (SC‐71; DSHB), and mouse IgM myosin heavy chain Type II B (BF‐F3; DSHB) were used as primary antibodies. Goat anti‐rat IgG Alexa Fluor‐488 (A11006; Invitrogen), goat anti mouse IgG2b Alexa Flour ‐350 (A21140; Invitrogen), goat anti‐mouse IgG Alexa Fluor Plus‐488 (A11001; Invitrogen), and goat anti‐mouse IgM Alexa Fluor‐555 (A21426; Invitrogen) were used as secondary antibodies. Following incubation with DAPI (62,248, Invitrogen) for 1 min, the sections were mounted using Fluorescent Mounting Medium (5570–0005, KPL). The CSA of the myofiber sections was measured from laminin‐α2‐stained images at 10× magnification. Images were acquired using an all‐in‐one fluorescence microscope (BZ‐X710) with a CFI Plan Fluor 10×/20× objective lens. (Keyence, Osaka, Japan). The deep learning algorithm CellPose was used to automatically segment the individual fibers and extract their contours (Stringer et al., 2021). Based on the extracted contours, the myofiber CSA was quantified using FIJI/ImageJ (Schindelin et al., 2012). In the analysis of muscle fiber types, fibers that were positive for both Type I and II A were classified as I/IIa hybrid, and fibers that were not stained for Type I, II A, or II B were classified as Type IIx.

### 2.5 Statistical analyses

Data are expressed as means ± SD. Two‐way ANOVA (Time × Treatment) was performed, followed by a Bonferroni multiple‐comparison test when an interaction was observed (Prism 5.0; GraphPad, La Jolla, CA). A two‐tailed unpaired *t*‐test was performed on CSA distribution of each timepoint. Statistical significance was defined as *p* < 0.05. The study design did not include blinding of the investigators or analysts. The datasets generated and/or analyzed during the current study are available from the corresponding author on reasonable request.

## 3 RESULTS

### 3.1 Body weight, skeletal muscle wet weight, and myofiber CSA

**FIGURE 1:**
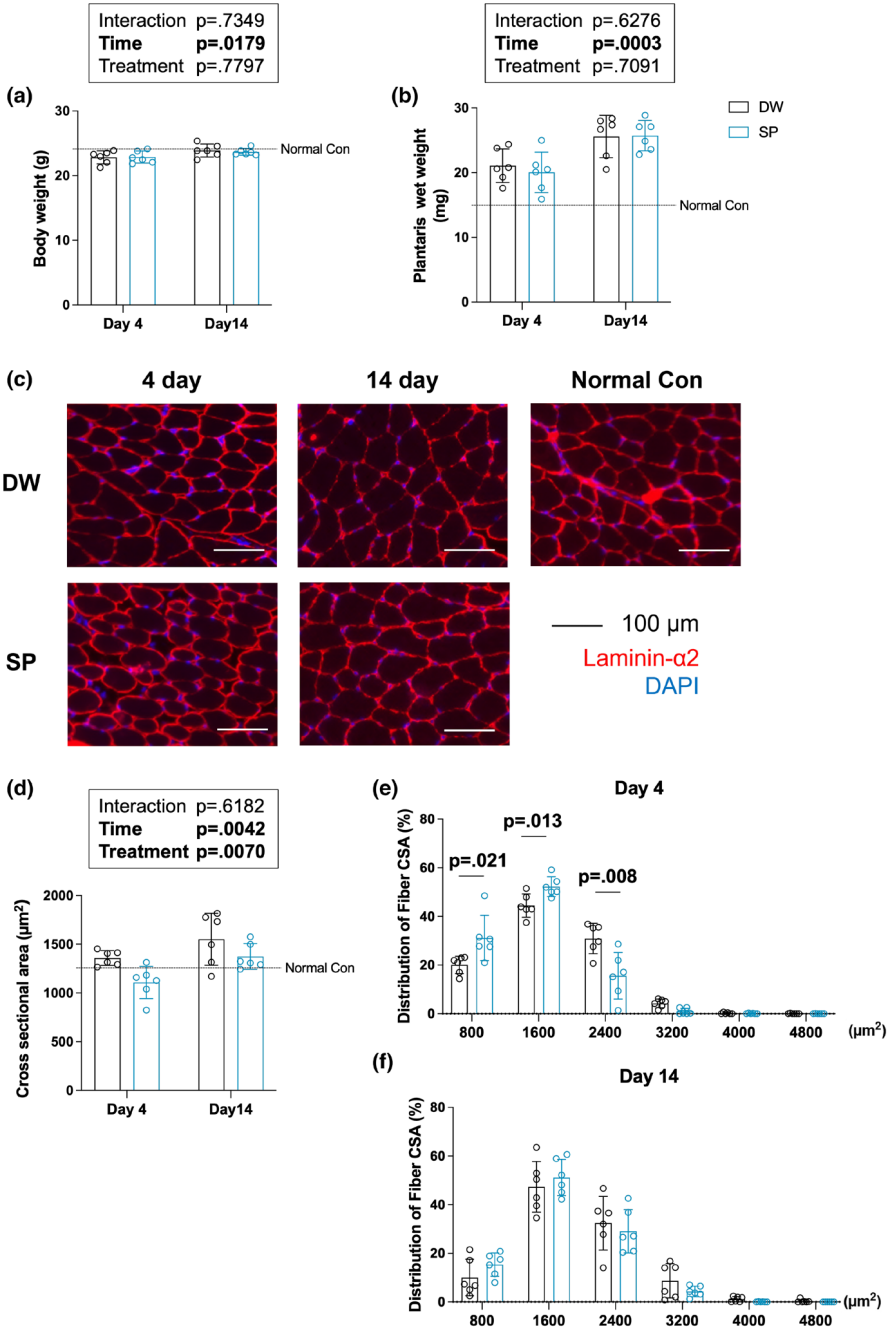
Body weight, muscle wet weight, and myofiber cross‐sectional area (CSA). (a) Body weight; (b) Plantaris wet weight; (c) representative immunohistochemical images; (d) mean fiber CSA; (e) fiber distribution at 4 days; (f) fiber distribution at 14 days. The data are presented in each plot as the mean ± standard deviation (n = 6 per group). DW, Distilled water group; SP, Spermidine group.

The effects of spermidine intake on body weight, skeletal muscle wet weight, and mean myofiber CSA were evaluated after overload‐induced skeletal muscle hypertrophy (Figure 1). Only the significant main effects of time were observed on body weight (*p* = 0.0179) and plantaris muscle wet weight (*p* = 0.0003) (Figure 1a,b). The significant main effects of time (*p* = 0.0042) and treatment (*p* = 0.0070) were observed on myofiber mean CSA (Figure 1c,d). The frequency distribution of the myofiber CSA at each time point is shown in Figure 1e,f. On day 4, spermidine intake significantly increased the frequency of myofiber CSA of 0–800 μm^2^ (*p* = 0.021) and 800–1600 μm^2^ (*p* = 0.013), and significantly decreased the frequency of myofiber CSA of 1600–2400 μm^2^ (*p* = 0.008) (Figure 1e). After 14 days, spermidine intake did not affect the frequency of myofiber (Figure 1f).

### 3.2 Skeletal muscle fiber type

**FIGURE 2:**
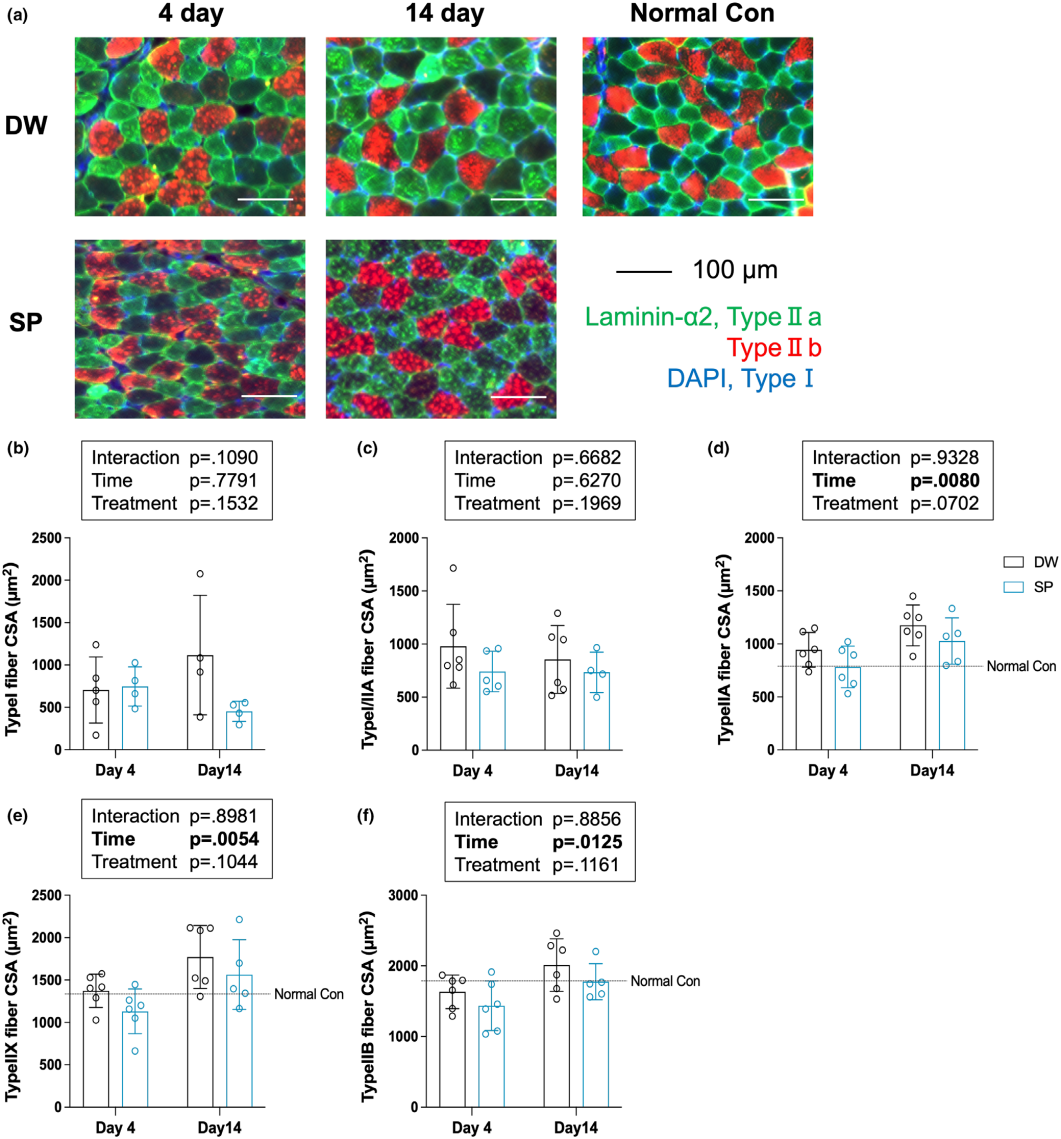
Muscle fiber type. (a) Representative immunohistochemistry images; (b) type I fiber CSA; (c) type I/IIa fiber CSA; (d) type IIa fiber CSA; (e) type IIx fiber CSA; and (f) type Ib fiber CSA. The data are presented as the mean ± standard deviation (n = 6 per group). DW, Distilled water group; SP, Spermidine group.

To determine the effect of spermidine intake on skeletal muscle fiber type after overload‐induced skeletal muscle hypertrophy, immunofluorescence staining was performed (Figure 2). The type I, type IIa and type IIb fibers were stained by immunofluorescence and classified into type l, type I/IIA hybrid, type IIa, type IIx and type IIb fibers. The myofiber mean CSA of each muscle fiber type are shown. The significant main effect of time were observed on type IIa, type IIx and type IIb (Figure 2a,d–f). No significant main effects or interactions between time and treatment were observed on type l, type I / IIA hybrid (Figure 2a–c).

### 3.3 Autophagy‐related proteins

**FIGURE 3:**
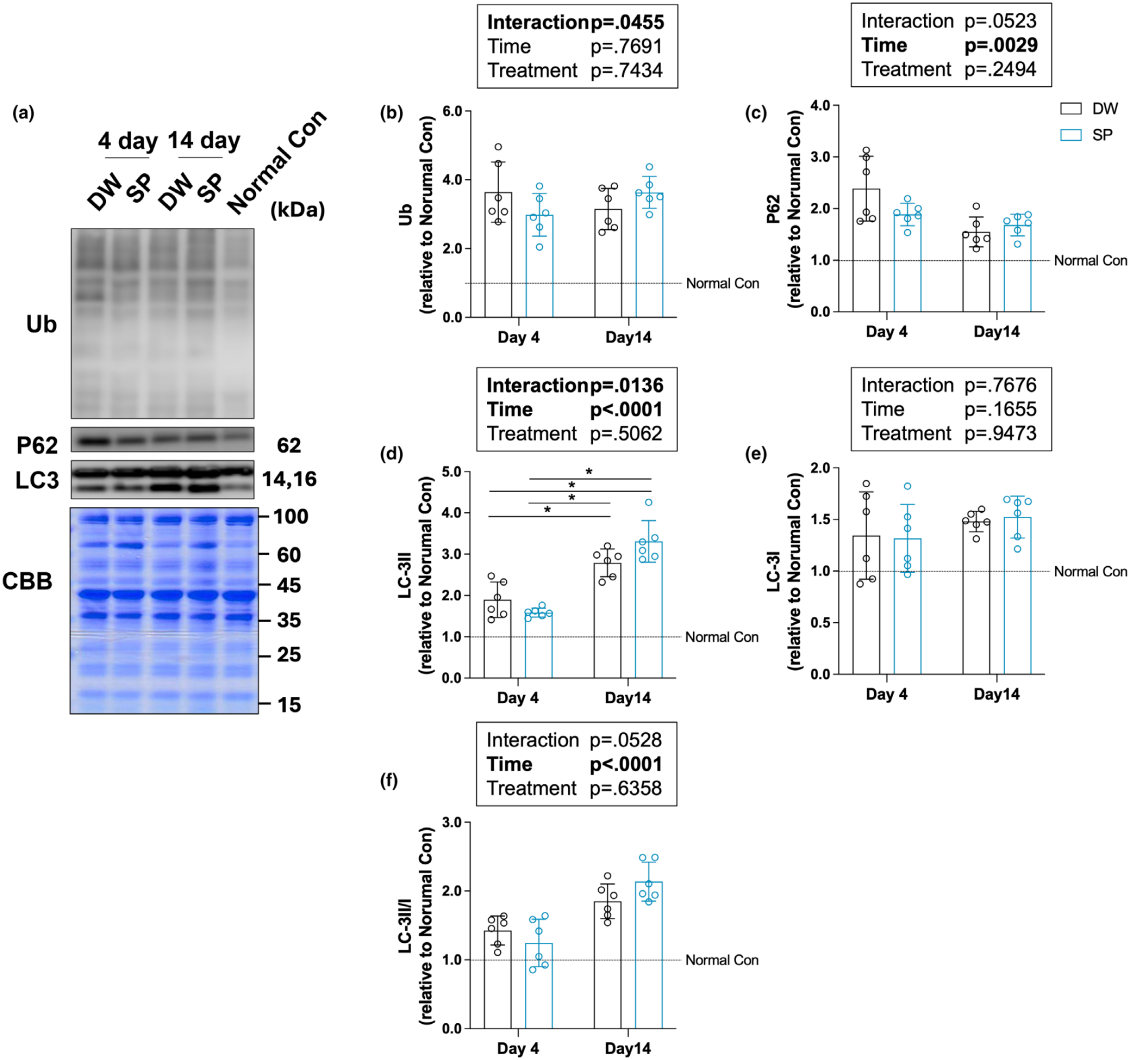
Autophagy‐related proteins. (a) Representative images of the bands and Coomassie brilliant blue staining of the total amount of protein; (b) ubiquitinated proteins; (c) p62; (d) LC3‐I; (e) LC3‐II; and (f) LC3‐II/I. The data are presented in each plot as the mean ± standard deviation (*n* = 6 per group). DW, Distilled water group; SP, Spermidine group. *p < 0.05.

To determine the effect of spermidine intake on autophagy after overload‐induced skeletal muscle hypertrophy, autophagy‐related proteins were analyzed by western blot analysis (Figure 3) The protein expression levels of LC3 I and II (markers for phagophores and autophagosomes, respectively), ubiquitinated proteins (damaged proteins), and P62 (autophagy substrates) were examined. Only the significant main effects of time were observed on P62 (*p* = 0.0029) and LC3 II/I (*p* < 0.0001) (Figure 3a,c,f). Only the significant interaction (*p* = 0.0455) were observed on Ub, but post hoc tests did not detect significant differences between all groups (Figure 3a,b). The significant main effects of time (*p* < 0.0001) and interaction (*p* = 0.0136) were observed on LC3 II (Figure 3a,d). The post hoc tests detected significant differences between Day 4 DW and Day 14 DW (*p* = 0.0028), between Day 4 DW and Day 14 SP (*p* < 0.0001), between Day 4 SP and Day 14 DW (*p* = 0.0001), and between Day 4 SP and Day 14 SP (*p* < 0.0001). No significant main effects or interactions between time and treatment were observed on LC3 I (Figure 3a,e).

### 3.4 mTOR signaling

**FIGURE 4:**
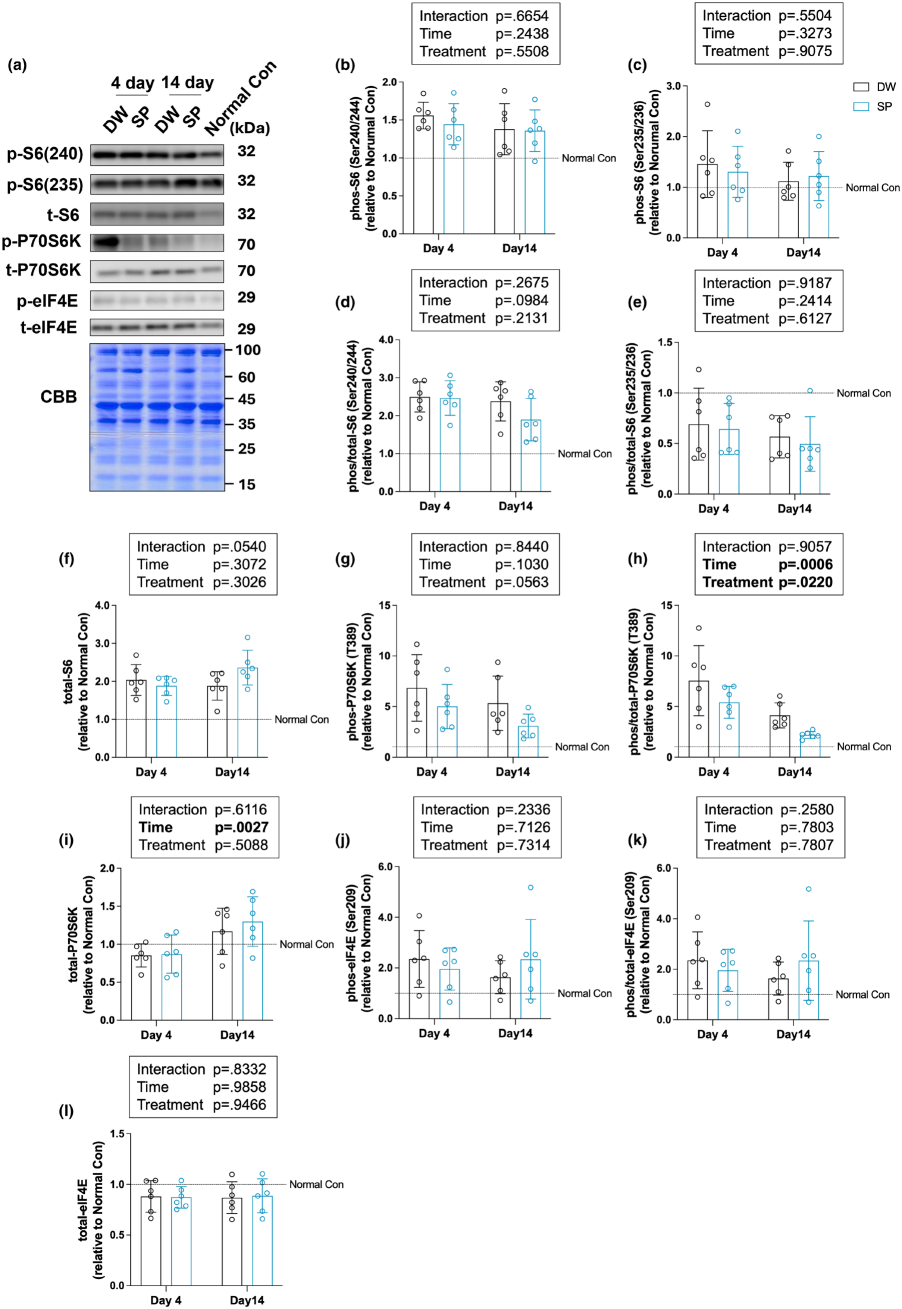
mTOR signaling. (a) Representative image of the bands and Coomassie brilliant blue staining of the total amount of protein; (b) p‐S6 (Ser240/244); (c) p‐S6 (Ser235/236); (d) p/t‐S6 (Ser240/244); (e) p/t‐S6 (Ser235/236); (f) t‐S6; (g) p‐P70S6K; (h) p/t‐P70S6K; (i) t‐P70S6K; (j) p‐eIF4E(Ser209); (k) p/t‐ eIF4E(Ser209); and (l) t‐eIF4E. The data are presented as the mean ± standard deviation (*n* = 6 per group). DW, Distilled water group; SP, Spermidine group.

To determine the effect of spermidine intake on mTOR signaling after overload‐induced skeletal muscle hypertrophy, we measured the phosphorylation levels of P70S6K, S6, and eIF4E, the downstream proteins of mTOR signaling, by western blot analysis (Figure 4). The significant main effect of time (*p* = 0.0006) and treatment (*p* = 0.0220) was observed on p−/t‐P70S6K (Figure 4a,h). The significant main effect of time (*p* = 0.0027) was observed on t‐P70S6K (Figure 4a,i). No significant main effects or interactions between time and treatment were observed on all other proteins (Figure 4a–gj–l).

### 3.5 Mitochondria‐related proteins

**FIGURE 5:**
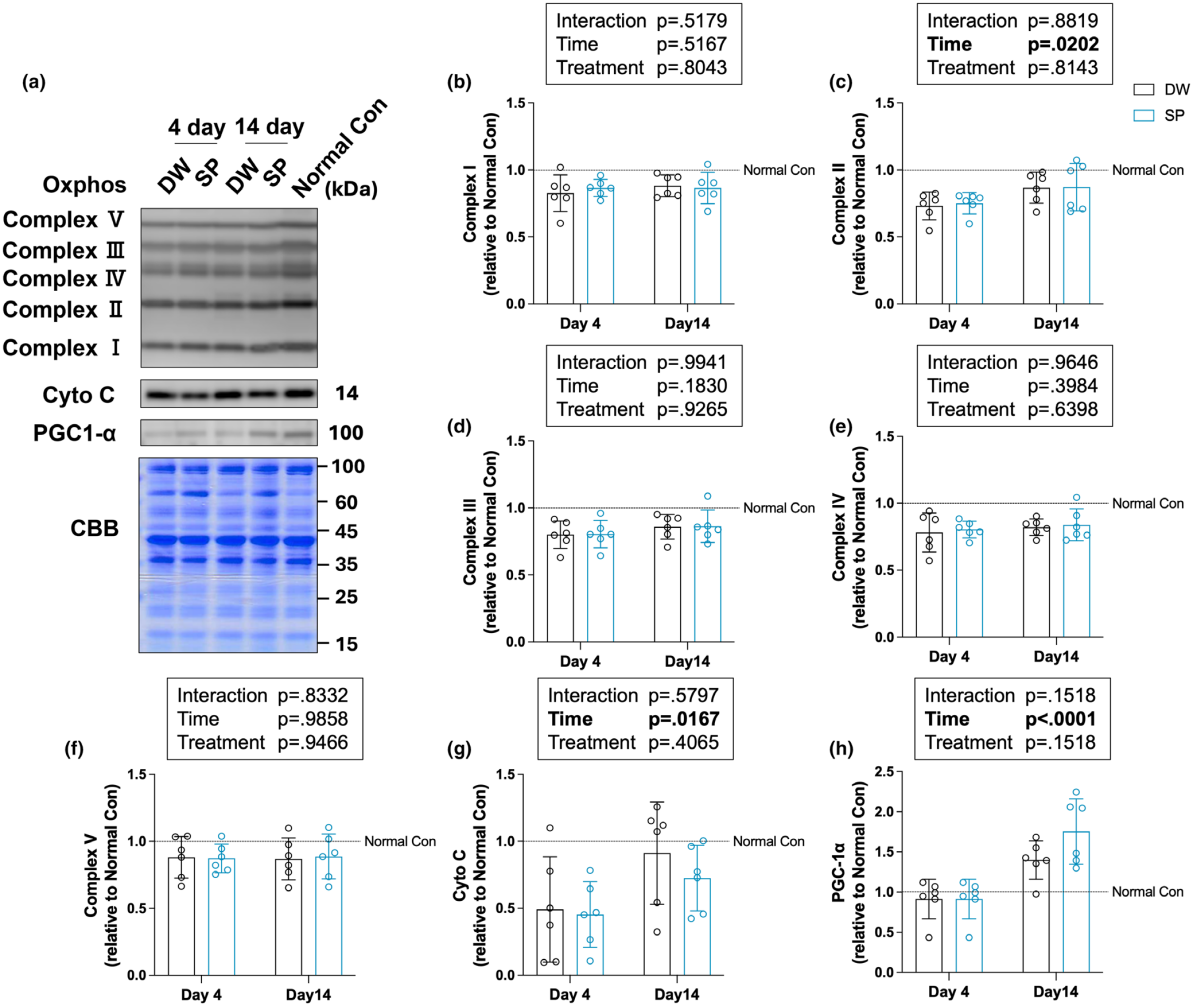
Mitochondria‐related proteins. (a) Representative images of the bands and Coomassie brilliant blue staining of the total amount of protein; (b) Complex I; (c) Complex II; (d) Complex III; (e) Complex IV; (f) Complex V; (g) Cytochrome C; and (h) PGC‐1α. The data are presented in each plot as the mean ± standard deviation (*n* = 6 per group). DW, Distilled water group; SP, Spermidine group.

To evaluate the effects of spermidine intake on mitochondria after overload‐induced skeletal muscle injury, mitochondria‐related proteins were analyzed by western blot analysis (Figure 5). We analyzed the protein expression levels of Oxphos (NDUFB8, SDHB, UQCRC, MTCO1, ATP5A), Cytochrome C (a protein responsible for ATP production through mitochondrial oxidative phosphorylation), and PGC‐1α (the master regulator of mitochondrial biogenesis). The significant main effect of time was observed for Complex II (*p* = 0.0202), CytoC (*p* = 0.0167), and PGC‐1α (*p* < 0.0001) (Figure 5a,c,g,h). No significant main effects or interactions between time and treatment were observed on all other proteins (Figure 5a,b,d–f).

### 3.6 Inflammatory cytokines

**FIGURE 6:**
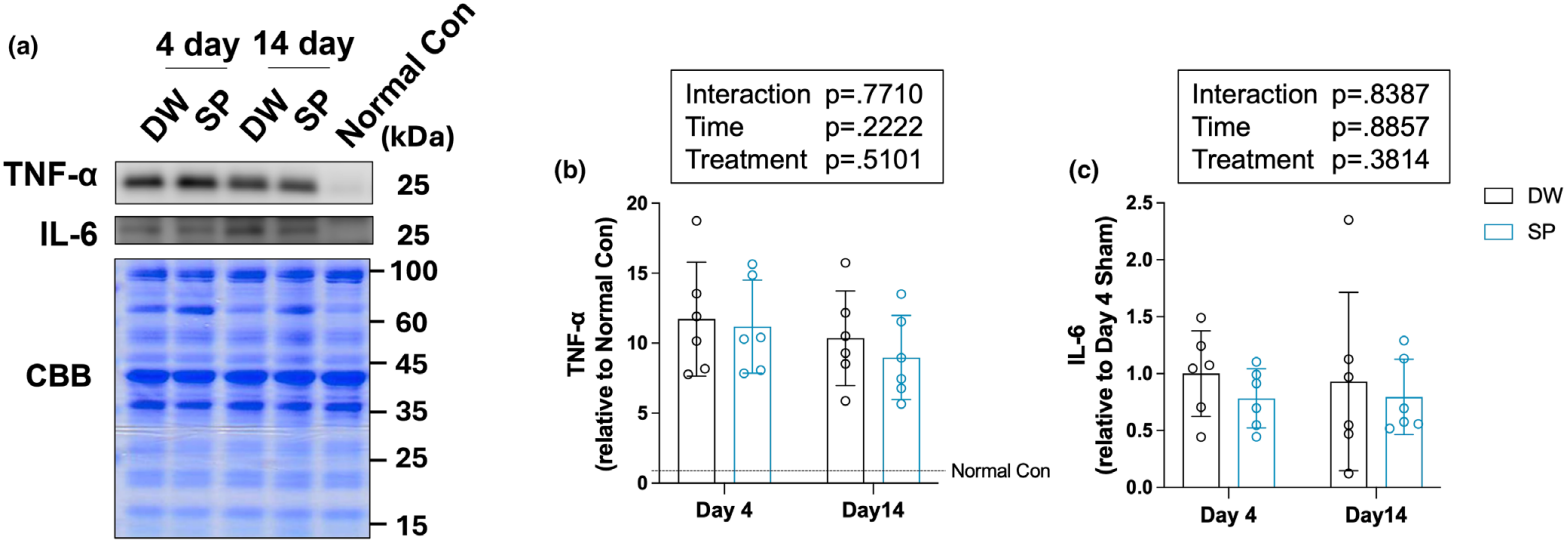
Inflammatory cytokines. (a) Representative images of the bands and Coomassie brilliant blue staining of the total amount of protein; (b) TNF‐α; (c) IL‐6. The data are presented as the mean ± standard deviation (*n* = 6 per group). DW, Distilled water group; SP, Spermidine group.

To determine the effect of spermidine intake on inflammation after overload‐induced skeletal muscle injury, inflammatory cytokines were analyzed by western blot analysis (Figure 6). No significant main effects or interactions between time and treatment were observed on TNF‐α and IL‐6 (Figure 6a–c).

## 4 DISCUSSION

Our results showed no significant change in skeletal muscle wet weight with spermidine intake. We also demonstrated that spermidine intake significantly decreased the mean fiber CSA. Moreover, spermidine intake significantly increased relative frequency of small myofiber and significantly decreased relative frequency of larger myofiber. However, spermidine intake did not significantly affect the CSA distribution day 14. Furthermore, spermidine intake did not affect the mean myofiber CSA of each muscle fiber type. We also analyzed autophagy, mTOR signaling, inflammation, and mitochondria. However, no significant effect of spermidine intake was observed on the expression levels of any proteins other than p−/t‐P70S6K. Although spermidine intake affected myofiber CSA, the discrepancy with skeletal muscle wet weight and lack of effect on the expression levels of the measured key proteins suggested that spermidine intake did not affect overload‐induced skeletal muscle hypertrophy, or if it did, its effect was limited and suppressive.

Spermidine intake significantly decreased the mean myofiber CSA. Previous studies have reported that spermidine promotes autophagy through the suppression of mTOR signaling (Pietrocola et al., 2015). We also demonstrated that spermidine intake significantly decreased p−/t‐P70S6K. Our results suggest that spermidine attenuates the skeletal muscle hypertrophy response by suppressing mTOR signaling. A systematic review of the synergistic muscle ablation model used in this study shows that the increase in plantaris muscle wet weight progressed linearly until day 15, and the data stabilized after this period (Terena et al., 2017). Therefore, it is possible that the changes of CSA distribution were only observed on day 4, because muscle hypertrophy was close to a plateau on day 14. However, the effect of spermidine intake on most of the proteins measured in this study was not significant. Therefore, the effect of mTOR inhibition on muscle hypertrophy is little or partial, and should be interpreted with caution.

In our previous study, spermidine intake increased the mean fiber CSA only 14 days after chemically induced skeletal muscle injury, suggesting that spermidine intake may promote the anabolic growth of differentiated muscle. However, the results of the present study suggest that spermidine supplementation does not affect skeletal muscle hypertrophy and may even inhibit it. A recent study reported that platelet‐targeted gene deficiency inhibits platelet migration and delays muscle regeneration (Graca et al., 2023). The aforementioned study and our previous study similarly found no difference in CSA 7 days after injury and only a significant difference in CSA 14 days following injury (Iwata et al., 2024). In a previous study examining the effect of icing on skeletal muscle regeneration, icing delayed changes in macrophages, stem cells, and inflammation during the early stages of injury, thereby reducing CSA 14 days after injury (Kawashima et al., 2021). Thus, it is possible that the effect on the regeneration process in the early stages of injury is reflected in CSA with a delay. Therefore, the effect on regeneration during the early stages of injury may be reflected in CSA with a delay. Although spermidine supplementation increased CSA at 14 days after injury in our previous study, this may not be the result of an acceleration of the process by which CSA increases in differentiated muscle. Instead, this may occur through the timing of the CSA increase, which influences the acute response after injury (Iwata et al., 2024). Spermidine has been reported to promote skin wound healing through the activation of the urokinase‐type plasminogen activator (uPA)/uPA receptor (uPAR) signaling pathway and induction of the inflammatory response (Ito et al., 2021). Therefore, spermidine may act on this pathway to promote regeneration at the early stage of injury.

In this study, we focused on the effects of spermidine, so we did not include a sham control group for any of the interventions. This may have resulted in us missing important effects of spermidine. It is necessary to examine the effects of spermidine intake on skeletal muscle in mice raised under normal conditions. In the overload‐induced muscle hypertrophy model used in this study, the skeletal muscle mass increased by approximately 1.5‐fold in 2 weeks (Terena et al., 2017). It has also been reported that despite the induction of skeletal muscle hypertrophy by synergist ablation, muscle strength per muscle CSA decreases (Atherton & Smith, 2012). Therefore, further studies are needed to clarify the effects of spermidine on muscle hypertrophy and increased strength due to resistance training in humans. This study did not confirm the general effects of spermidine as a calorie restriction mimetic on autophagy or mitochondria. It has been reported that when mice are allowed free access to water, they drink more than twice as much water during the dark period as during the light period (Costello et al., 2023). In this study, sampling was performed in the middle of the light period, so it is necessary to be aware that the acute effects of spermidine intake may have been underestimated. Furthermore, future studies should also determine the effects of spermidine on mitochondria in skeletal muscle hypertrophy by analyzing autophagy flux assays and mitochondrial quality (Kaizuka et al., 2016; Redmann et al., 2018; Ueno & Komatsu, 2020). The lack of blinding may have introduced potential bias in data collection or analysis, which should be considered when interpreting the results.

In conclusion, spermidine intake did not significantly affect the phenotype or protein levels related to autophagy, mitochondria, and inflammation at most time points examined in an overload‐induced skeletal muscle hypertrophy model.

## AUTHOR CONTRIBUTIONS

**Tomohiro Iwata:** conceptualization, investigation, validation, data curation, writing the original draft, and artwork; **Takanaga Shirai:** investigation, data curation, review and editing; **Riku Tanimura:** investigation; **Ryoto Iwai:** investigation; **Tohru Takemasa:** supervision, review, and editing; All authors approved the final version of the manuscript.

## FUNDING INFORMATION

This work was supported by JSPS KAKENHI Grant Number 23H03266, 23KJ2060, JST‐SPRING program, Leave a Nest Co., Ltd. incu ・ be award, and IKEDA SCIENTIFIC Co., Ltd. bridge fellowship.

## CONFLICT OF INTEREST STATEMENT

The authors declare that they have no known competing financial interests or personal relationships that could appear to have influenced the work reported in this article.
